# Spatio-Temporal Distribution of *Mycobacterium tuberculosis* Complex Strains in Ghana

**DOI:** 10.1371/journal.pone.0161892

**Published:** 2016-08-26

**Authors:** Dorothy Yeboah-Manu, P. Asare, A. Asante-Poku, I. D. Otchere, S. Osei-Wusu, E. Danso, A. Forson, K. A. Koram, Sebastien Gagneux

**Affiliations:** 1 Noguchi Memorial Institute for Medical Research, University of Ghana, Legon, Accra, Ghana; 2 Department of Chest Diseases, Korle-Bu Teaching Hospital, Korle-bu, Accra, Ghana; 3 Department of Medical Parasitology and Infection Biology, Swiss Tropical and Public Health Institute, Basel, Switzerland; 4 University of Basel, Basel, Switzerland; University of Minnesota, UNITED STATES

## Abstract

**Background:**

There is a perception that genomic differences in the species/lineages of the nine species making the *Mycobacterium tuberculosis* complex (MTBC) may affect the efficacy of distinct control tools in certain geographical areas. We therefore analyzed the prevalence and spatial distribution of MTBC species and lineages among isolates from pulmonary TB cases over an 8-year period, 2007–2014.

**Methodology:**

Mycobacterial species isolated by culture from consecutively recruited pulmonary tuberculosis patients presenting at selected district/sub-district health facilities were confirmed as MTBC by IS*6110* and rpo*ß* PCR and further assigned lineages and sub lineages by spoligotyping and large sequence polymorphism PCR (RDs 4, 9, 12, 702, 711) assays. Patient characteristics, residency, and risks were obtained with a structured questionnaire. We used SaTScan and ArcMap analyses to identify significantly clustered MTBC lineages within selected districts and spatial display, respectively.

**Results:**

Among 2,551 isolates, 2,019 (79.1%), 516 (20.2%) and 16 (0.6%) were identified as *M*. *tuberculosis* sensu stricto (MTBss), *M*. *africanum* (Maf), 15 *M*. *bovis* and 1 *M*. *caprae*, respectively. The proportions of MTBss and Maf were fairly constant within the study period. Maf spoligotypes were dominated by Spoligotype International Type (SIT) 331 (25.42%), SIT 326 (15.25%) and SIT 181 (14.12%). We found *M*. *bovis* to be significantly higher in Northern Ghana (1.9% of 212) than Southern Ghana (0.5% of 2339) (p = 0.020). Using the purely spatial and space-time analysis, seven significant MTBC lineage clusters (p< 0.05) were identified. Notable among the clusters were Ghana and Cameroon sub-lineages found to be associated with north and south, respectively.

**Conclusion:**

This study demonstrated that overall, 79.1% of TB in Ghana is caused by MTBss and 20% by *M*. *africanum*. Unlike some West African Countries, we did not observe a decline of Maf prevalence in Ghana.

## Introduction

One of the major threats to tuberculosis (TB) control is the emergence of strains that are resistant to most of the anti-TB drugs, which could make a treatable disease untreatable [1]. Other factors that limit current TB control efforts are lack of effective vaccines, lack of cheap but effective rapid diagnostics, emergence of HIV/AIDS pandemic and limited understanding of the diversity of circulating strains [1]. The increase in TB cases globally requires a concerted effort to control this global public health problem. This calls for improved understanding of the disease pathogenesis, epidemiology, and genetic variability within the causative agent.

TB is caused by a group of closely related acid-fast gram-positive bacteria, together referred to as the *Mycobacterium tuberculosis* complex (MTBC) [2, 3]. The MTBC comprises *M*. *tuberculosis* sensu stricto (MTBss), *M*. *Africanum* (Maf), *M*. *microti*, *M*. *bovis*, *M*. *caprae*, *M*. *mungi*, *M*. *suricattae*, *M*. *orygis* and *M*. *pinnipedii*. They have varying host ranges: *Mycobacterium microti* affects voles, [4, 5] *M*. *caprae* a pathogen of goats and sheep [6]. *M*. *mungi*: Mangoose pathogen, *M*. *orygis* a pathogen of antelope [7], *M*. *pinnipedii* a pathogen of seals and sea lions [8]. *Mycobacterium bovis* displays the broadest spectrum of host affecting humans and animals [9]. *Mycobacterium tuberculosis* sensu stricto and *M*. *africanum* are the main causative agents of TB in humans; referred to as human adapted MTBC and the remaining seven species as animal adapted [3]. The human adapted MTBC comprises seven main phylogenetic lineages, which have been confirmed by single nucleotide polymorphisms (SNPs) and whole genome sequencing [3, 10–12]. These lineages were further found to exhibit a phylogeographical structure, which means that specific lineages are closely associated with specific geographic regions, and preferentially infect persons originating from these regions. Importantly, findings from recent genomic analysis indicate that some of these human MTBC lineages are as genetically distinct from each other as from the animal-adapted forms of MTBC [10] and have genomic differences that may influence host-pathogen interaction as well as applicability of control tools such as diagnostics and vaccine. Thus the lineages distribution needs to be taken into account in the development and testing of new control tools such as vaccines to account for any possible differential phenotypes. West Africa shows a unique mycobacterial population structure, as it is the only region worldwide where lineages of Maf are endemic [13].

Work done mainly in the Gambia, suggested that Maf is attenuated compared to MTB [13, 14]. While both transmit equally, the rate of progression to disease was slower in Maf infected contacts. Furthermore, MTBC lineage 6 (also known as Maf West Africa 2) was found to be associated with HIV co-infection and reduced ESAT6 secretion [15, 16]. Thus MTBss seems to have a competitive advantage that could lead to a replacement of Maf with the more virulent MTB. This might be particularly likely due to the large population increases in West African cities [17]. One could also argue that with the HIV pandemic and other immune suppression diseases, Maf will still be an important pathogen in West Africa. Recent publications from various countries, however, observed an interesting trend: the slow replacement of Maf with MTBss, especially the Cameroun sub-lineage of lineage 4. This phenomenon was first described in Guinea-Bissau, where lineage 6 decreased from 51% to 39% in about 2 decades [18]. Declines in prevalence of the other *M*. *africanum* lineage have also been observed in Côte d’Ivoire, and Cameroon [19–22].

At the same time, understanding of the genetic population structure of circulating MTBC strains is increasingly becoming important for TB control. Current genomic studies have revealed that substantial strain genetic diversity exists among the different members and genotypes of MTBC, which may have implications for the development and deployment of new TB vaccines and diagnostics [23]. In this study, we analysed the distribution of MTBC lineages and sub-lineages in Ghana, a country harbouring six of the seven identified MTBC [24, 25] over an 8-year period. Our findings indicated a fairly constant distribution of the two main MTBC species and lineages over time. In addition, we observed clustering of some MTBC lineages at specific geographical locations.

## Materials and Methods

### Ethics Statement

The Scientific and Technical Committee and then the Institutional Review Board (IRB) of the Noguchi Memorial Institute for Medical Research with a federal wide assurance number FWA00001824 reviewed the protocols and procedures for this study and approved them. Written informed consent was obtained from participants using a designed form which was approved by the IRB. Methods for sputum sampling conformed to WHO guidelines (two sputa per patient) and patients’ identity was protected.

### Study Locale and Participants Data

**Fig 1:**
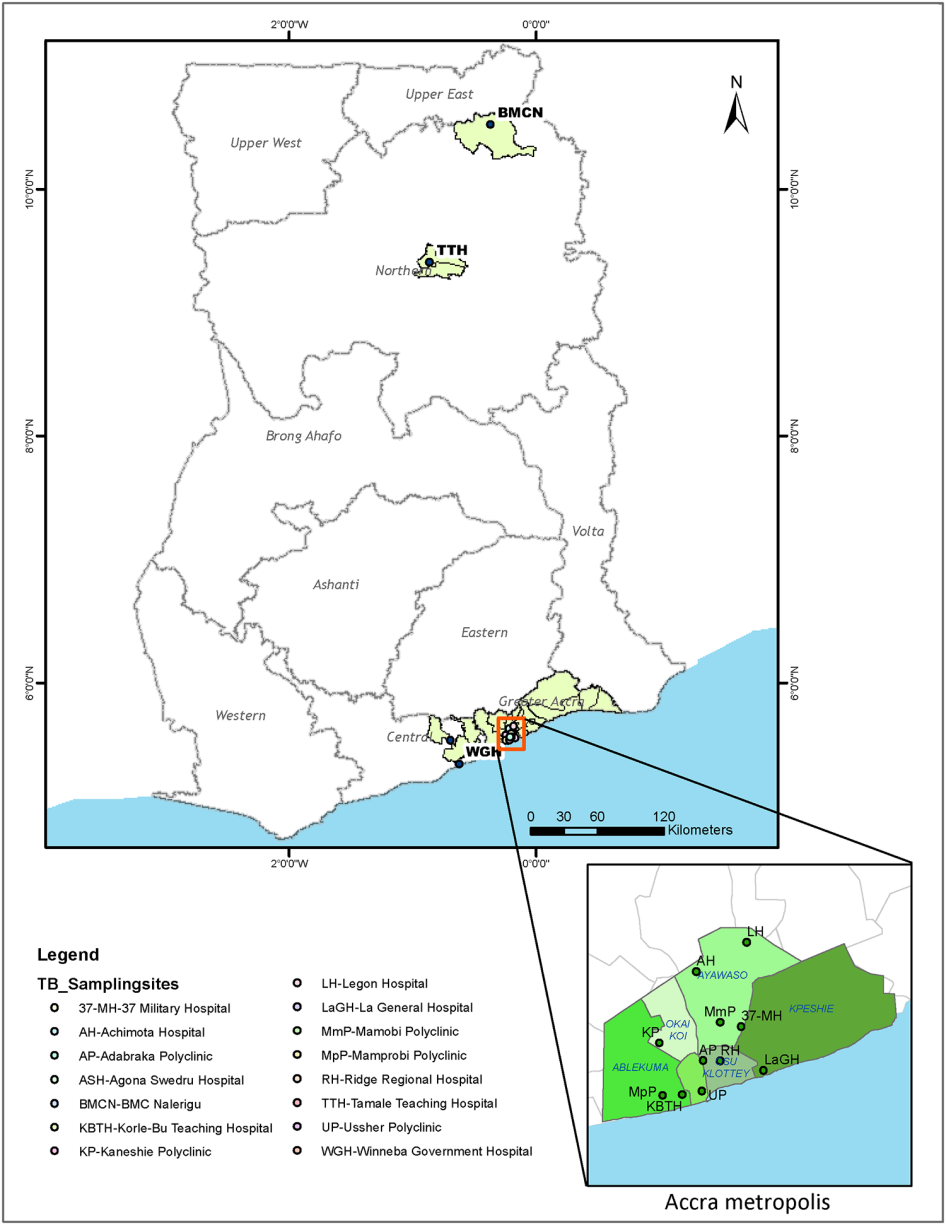
Map of sampling sites and study area. Sputum samples were obtained from fifteen sampling sites (health facilities) all located within three regions in Ghana; Greater Accra, Central and Northern regions. During the period September 2012 –December 2014, samples were obtained mainly from all the 13 diagnostic centres within the Accra metropolis, (serving more than 46% of the Greater Accra region populace) and the two health facilities located in the northern region (Tamale Teaching Hospital and BMC Nalerigu). The ArcMap program in ArcGIS v. 10.0 was used to create the map.

The study was conducted from July 2007 to December 2014 in Ghana, involving sputum smear positive TB cases. From July 2007 to December 2011, patients were recruited from five health facilities; Korle-Bu Teaching Hospital (KBTH) in the Greater Accra region, Agona Swedru Government Municipal Hospital (ASH), Winneba Government Hospital (WGH), St Gregory Catholic Clinic from the Central Region, all in the southern section of Ghana (Fig 1). Between 2012 and 2014, based on an on-going prospective study; sputum was collected from suspected TB cases reporting to the selected health facilities in the Accra Metropolitan Authority (AMA) and 2 districts (Mamprusi East (MamE) and Tamale Metropolis (TamM)) in the northern region of Ghana (Fig 1) after informed consent. The study sampling sites span 13 administrative districts with a combined population of 4,024,810 [26–29] in three regions according to the current administrative district division status created in 2013 (S1 Table). The AMA administrative district is made up of 10 sub-districts; Ablekuma South, Ablekuma North, Ablekuma Central, Ashiedu Keteke, Okai Koi South, Okai Koi North, Osu Klotey, Ayawaso East, Ayawaso Central and Ayawaso West Wogon with a combined population of 1,665,086 according to the 2010 population and housing census conducted in Ghana [26, 30]. The ten sub-districts within the AMA for the purposes of this study were merged into 5 sub-districts (Fig 1), based on the geographical demarcation existing as at 2007. These 5 sub-districts were: Ablekuma (Able), Ashiedu Keteke (AshK), Ayawaso (Ayaw), Okaikoi (Okai) and Osu Klottey (OsuK). The AMA covers a total land area of 136.674 square kilometres. Kpeshi, a former administrative sub-district of AMA is located on the eastern boundary of AMA and has been currently broken down into two districts; La Dade Kotopon Municipal and Ledzokuku/Krowor Municipal. We included the former Kpeshie demarcation in all targeted analysis involving AMA as TB patients still access facilities within AMA. Sites in the northern region, TamM and MamE, covers a land area of 790.5 and 1,823.6 square kilometres respectively with a combined population of 492,360 (TamM: 371,351 and MamE: 121,009). Together, TamM and MamE constitute 19.9% of the total population in the northern region of Ghana [29].

Information on age, sex, nationality, ethnicity, employment status, previous history of TB, crowding, substance abuse and duration of symptoms were obtained from the patients with a structured questionnaire.

### Mycobacterial Isolation, Species and Lineage Classification

Mycobacterial species were isolated by decontaminating sputum samples with equal amount of 5% oxalic acid solution, then inoculation on Lowenstein Jensen media and incubated as previously described [31]. Members of the MTBC were confirmed by PCR detection of the insertion sequence IS*6110* and rpoβ as previously described [32]. Classification into the main phylogenetic lineages was achieved by large sequence polymorphism typing assay identifying regions of difference (RD) 4, 9, 12, 702, 711 [2, 3, 14] and also by spoligotyping following manufacturer’s directions (Isogen Bioscience BV Maarssen, The Netherlands).

### Data Management and Analysis

Data obtained using the structured questionnaire was double entered using Microsoft Access and validated to correct entry errors. The questionnaire data primarily provided us with the year of diagnosis and residential address (location) of each TB case for the spatio-temporal analysis. In addition to these data, other demographic and clinical characteristics of each participant as indicated above were generated. The association of specific lineages and/or sub-lineages of the MTBC with time and/or geographical locations were explored with Fishers exact test using the Stata statistical package (Stata Corp., College Station, TX, USA). All analyses were run with significance level pegged at p < 0.05.

To determine the TB case notification rates for the period 2012–2014, we obtained the projected population of the individual districts using the exponential growth rate formula; **P**_**t**_ **= P**_**o**_**e**^**rt**^ (based on the assumption of constant population growth similar to compounded interest) [33]. Where; P_t_ = projected population, P_o_ = initial population, e = base of the natural logarithm, r = intercensal growth rate and t = time elapsed after last census. The intercensal growth rates (S1 Table) used for the various regions were obtained from the Ghana statistical service 2010 population and housing census data [26].

The GIS co-ordinates of the participants’ self-reported district of residency was used to construct a pictorial plot of the distribution of the MTBC lineages analysed using the ArcMap (Economic and Social Research Institute, version 10.0) [34]. The district allocation data generated was linked to a molecular data of all TB isolates and was used for TB lineage clustering analysis.

### Spatial and Space-time Analysis

Kulldorff’s scan statistics (SaTScan^™^ 9.4.2) tool [35], a commonly used tool for spatial and space-time cluster analysis for diseases in a wide variety of settings [36–40] was used for analysis of spatio-temporal clustering of TB cases using data obtained only within the time period; September 2012 to December 2014. TamM was excluded from analysis where 2012 data was used since we recorded no TB case in 2012. The Kulldorff’s scan statistics tool was used to detect significant MTBC clusters using the Monte Carlo simulations [41]. Three input files (cases, population and coordinates) were built using excel and saved in the required format for upload into the SaTScan software. The discrete Poisson model was used for the analysis with the assumption that the number of cases at each district had Poisson distribution with a known population at risk [41]. All other parameters were set at default for both spatial and space-time analysis (S4 Table). The results of the analyses were tabulated to add statistical significance to the inferences made using ArcMap.

### Normalization of TB cases for within district comparison

To analyse the spatial and space-time distribution of MTBC cases at the district/sub-district level, we normalized the relative case frequencies against their respective reference population obtained from the Ghana Statistical Service [26, 27, 29, 30] Also, records of specific genotypes (or sub-lineages) were normalized using all recorded cases of the specified genotype (or lineage) within the specified time. For example, all Ghana and Cameroon sub-lineages per district were normalized using all Lineage 4 cases as the denominator.

## Results

### Characteristics of Patients Presenting with Tuberculosis

**Fig 2:**
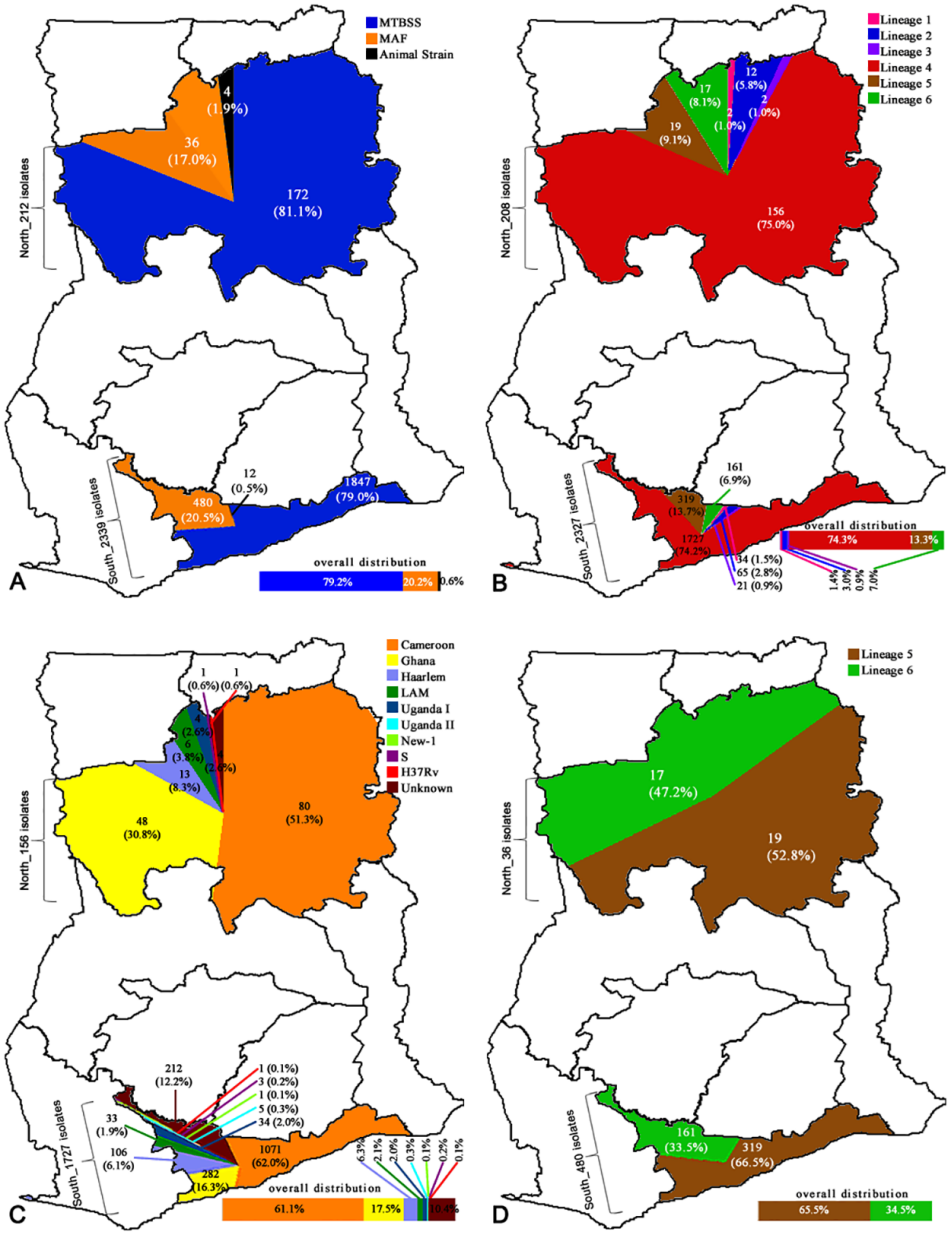
Spatial distribution and prevalence of identified *mycobacterium tuberculosis* lineages. Diagram shows the spatial distribution of (A) 2551 *Mycobacterium tuberculosis* complex (MTBC) strains; (B) 2535 human adapted MTBC; (C) regional prevalence of 1883 Lineage 4 sub lineages; (D) regional prevalence of 516 *Mycobacterium africanum* (Maf) isolates from the geographical regions served by the health facility where sampling was carried out in Ghana. Animal strains were found to be associated with the North (p = 0.0389). Similarly, Lineage 2 was found to be associated with the North (p = 0.0006). The most dominant Lineage 4 sub-lineage in the North is Ghana (p = 0.0000) whereas in the South is Cameroon, even though the association is not statistically significant. The unknown sub-type of Lineage 4 is associated with South (p = 0.0001).

Sputum smear positive patients from whom MTBC strains were isolated were 2551/3110 (82.0%) cases, comprising 70% (1789/2551) males and 30% (762/2551) females. Participants’ age ranged between 2 to 91 and a median age of 39 years. Ninety-one point seven percent (2339/2551) of the patients were from Southern Ghana and the remaining 8.3% (212/2551) from Northern Ghana (Fig 2). The HIV status of 1613 patients was indicated, of which 15.5% (250/1613) were HIV positive. The additional demographic and clinical characteristics of the cases are indicated in Table 1.

**Table 1:** Demographic and clinical data of 2551 TB cases.

Variable (Total number analysed)	Number (Percentage)
***Sex* (2551)**	
Male	1789 (70.1)
Female	762 (29.9)
***Age category* (2551)**	
08–25	459 (18.0)
26–40	1082 (42.4)
41–77	980 (38.4)
>77	30 (1.2)
***Residency* (2551)**	
North	212 (8.3)
South	2339 (91.7)
***Occupation* (2530)**	
Skilled	490 (19.2)
Unskilled	1890 (74.1)
Unemployed	150 (5.9)
***Settlement* (2550)**	
Urban	2283 (89.5)
Rural	267 (10.5)
**HIV status**^1^ **(1613)**	
Yes	250 (15.5)
No	1363 (84.5)
**Presence of BCG scar** ^2^**(1817)**	
Yes	904 (49.8)
No	913 (50.2)
Income* **(2551)**	
None	771 (30.2)
Low	1500 (58.8)
High	280 (11.0)
**Drinking status**^3^ **(2071)**	
Yes	556 (26.8)
No	1515 (73.2)
**TB in the past**^4^ **(2029)**	
Yes	218 (10.7)
No	1811 (89.3)
**Education Level (2551)**	
None	300 (11.7)
Primary	605 (23.7)
Secondary	1551 (60.7)
Tertiary	95 (3.7)
**Smear Grade (2551)**	
Scanty	235 (9.2)
1	985 (38.6)
2	531 (20.8)
3	800 (31.4)
**In Household number (2551)**	
<5	589 (23.1)
>5	1962 (76.9)
**Ethnicity (2551)**	
Akan	800 (31.4)
Ewe	339 (13.3)
Ga	595 (23.3)
Mole -Dagbon	36 (1.4)
Gruma	5 (0.2)
Guan	9 (0.4)
Others	767 (30.0)
**Marital status (2551)**	
Single	859 (33.7)
Married	1037 (40.7)
Divorced	221 (8.7)
Widowed	104 (4.1)
Co habiting	330 (12.9)
**Cough (2551)**	
< 2 weeks	2117 (82.9)
> 2 weeks	122 (4.8)
Symptoms other than cough	312 (12.2)
**Night Sweat** ^5^ **(2219)**	
Yes	1372 (61.8)
No	847 (33.2)
**Hemoptysis** ^6^ **(2227)**	
Yes	480 (21.6)
No	1747 (78.4)
**House type (2551)**	
Self-contained	486 (19.1)
Compound House	1707 (66.9)
Others	358 (14.0)
**Swollen glands** ^7^ **(2211)**	
Yes	210 (9.5)
No	2001 (91.5)
**Chest Pain** ^8^ **(2234)**	
Yes	1717 (76.9)
No	517 (23.1)
**Nationality** ^9^ **(2251)**	
Ghana	2187 (97.2)
Nigeria	21 (0.9)
Togo	12 (0.5)
Niger	11 (0.4)
Ivory Coast	7 (0.3)
Others West African Nationals	13 (0.7)
**Contact with TB patient (2551)**	
Yes	316 (12.4)
No	2234 (87.6)
**Smoking** ^10^**(2419)**	
Yes	500 (20.7)
No	1919 (75.3)

^1^ = 938 missed data for HIV status,

^2^ = 734 missed data for Presence of BCG scar,

^3^ = 480 missed data for Drinking status,

^4^ = 522 missed data for TB in the past,

^5^ = 332 missed data for Night Sweat,

^6^ = 324 missed data for Night Sweat,

^7^ = 340 missed data for Swollen glands,

^8^ = 317 missed data for Chest Pain,

^9^ = 300 missed data for Nationality,

^10^ = 132 missed data for Smoking.

* Income below 1000GH₵ was defined as low whilst those above 1000GH₵ as high.

### The Population Structure of MTBC causing pulmonary TB in Ghana

Two thousand six hundred and three mycobacterial isolates were obtained from 3110 samples giving a cumulative isolation rate of 83.7%. We identified 2551 of the isolates as members of the MTBC and 52 as non-tuberculous mycobacteria (NTM) (as well as those with negative mycobacteria) which were excluded from further analysis. Among those confirmed as MTBC, 2019 (79.1%) were MTBss, 516 (20.2%) were Maf, and 16 (0.6%) animal strains (15 *M*. *bovis* (SIT 1037, 482*)* and 1 *M*. *caprae*) (Fig 2A). Six of the seven lineages of the human adapted MTBC (Maf and MTBss) were identified in the following proportions: L1 (36; 1.4%), L2 (77; 3.0%), L3 (23; 0.9%), L4 (1883; 74.3%), L5 (338; 13.3%) and L6 (178; 7.0%), respectively (Fig 2B). The sub-lineages identified within the L4 were the Cameroon (1151:61.1%) followed by the Ghana (330; 17.5%), then Haarlem (119; 6.3%), LAM (39; 2.1%), Uganda I (38; 2.0%), Uganda II (5; 0.3%), New-1 (1; 0.1%), S (3; 0.2%) and H37Rv-like (2; 0.1%) (Fig 2C).

### Spatial Distribution of MTBC Genotypes

The combined number of isolates analysed from the different geographical areas, identified species, lineages and sub-lineages are indicated in Fig 2A–2D, respectively. As shown in Fig 2D, there was no statistical difference in the Maf proportion between the north (17.0%; 36/212) and the south (21.9%; 378/1726) (p = 0.1099). However, we found the proportion of animal-adapted species (MTBC other than MTBss and Maf) in the north (1.9%; 4/212) to be more than twice the proportion in the south (0.7%; 12/1726) (p<0.0884; OR = 2.74). There was unequal spatial distribution of L4 sub-lineages and spoligotypes. The proportion of the Ghana sub-lineage was statistically higher in Northern Ghana (32.3%) compared to 20.1% in the south (p = 0.0016, OR = 1.9, 95%CI = 1.3–2.9). Whereas the Spoligotype international type (SIT) 61 was more likely to be found in the south (p = 0.0330; OR = 0.7; 95% CI = 0.4–0.9), the SIT 53 was more likely to be found in the north (p = 0.0015; OR = 2.0; 95% CI = 1.3–3.1). In addition, L2 was proportionally higher in the north (5.7%; 12/212) compared to the south (3.1%; 53/1726).

**Fig 3:**
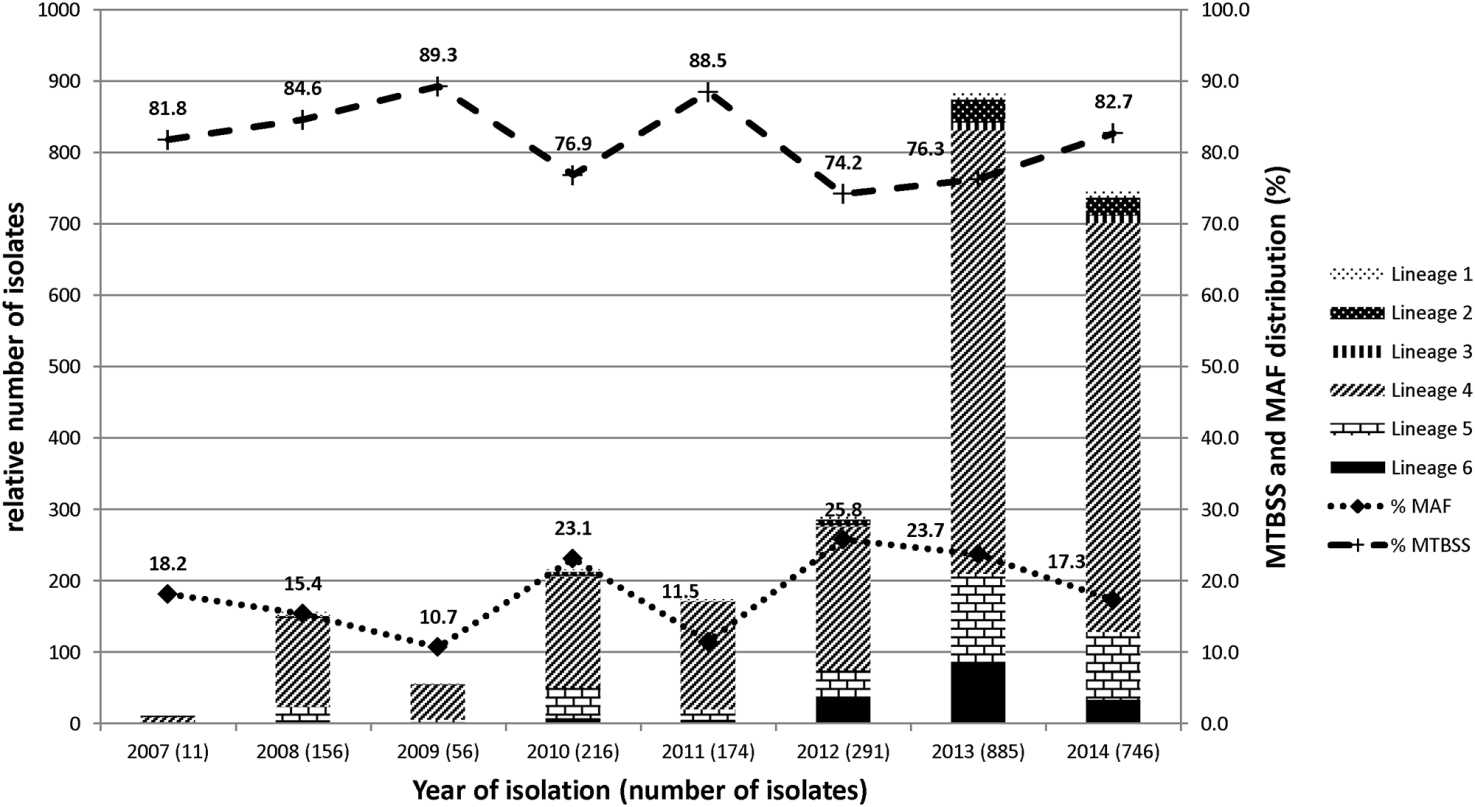
Temporal distribution and prevalence of human adapted *mycobacterium tuberculosis* complex (MTBC). Figure displays a stacked graph showing the temporal distribution of human adapted MTBC (left y-axis) and a linear graph showing the prevalence of *Mycobacterium tuberculosis* sensu stricto (MTBss) and *Mycobacterium africanum* (Maf) (right y-axis) over the entire 8-year study period.

**Fig 4:**
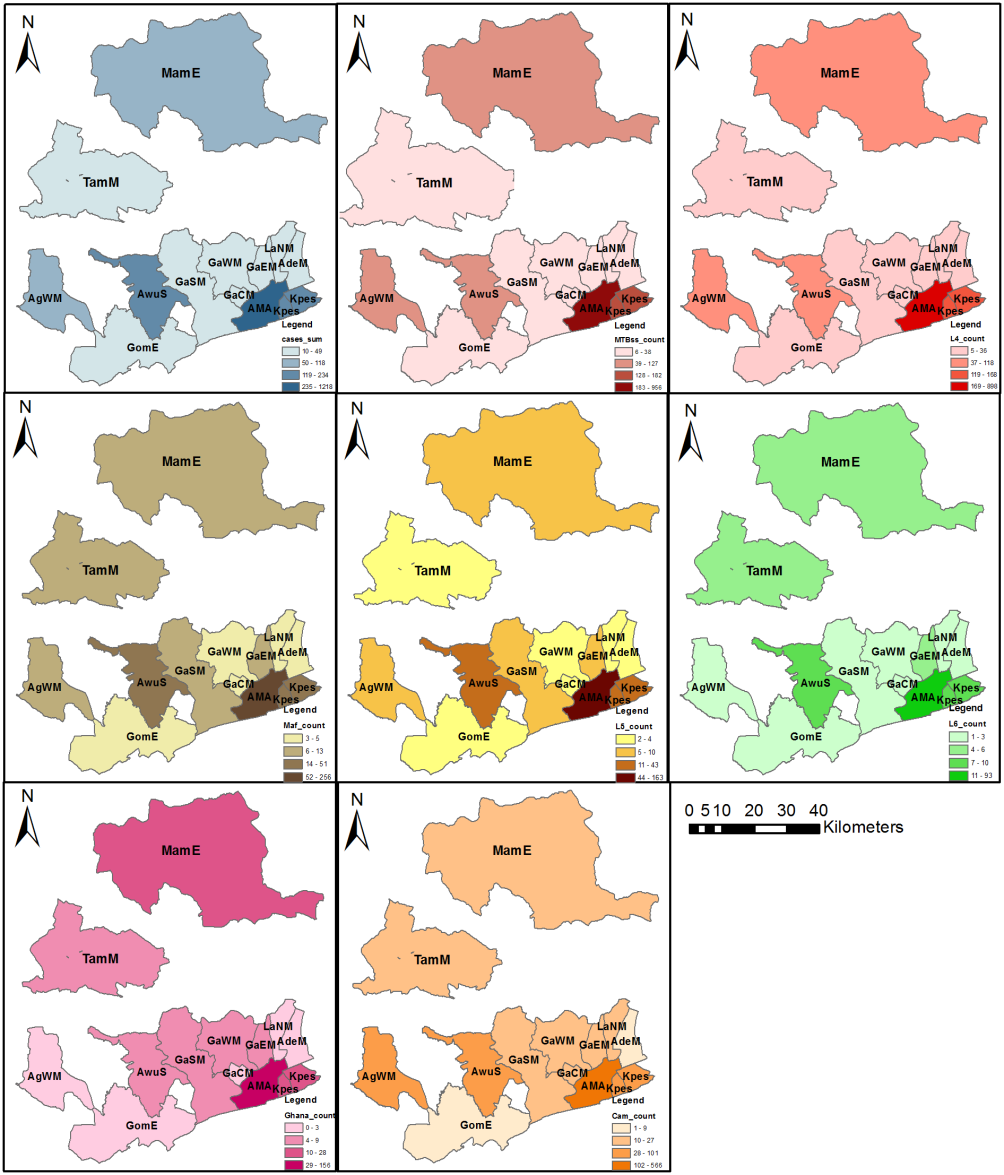
Spatial distribution of human adapted MTBC lineages and major sub-lineages within the eight-year study period. The figure shows the distribution of MTBC species/lineages/sub-lineages within the 13 districts where participants resided. The blue coloured panel shows the distribution of all the tuberculosis cases recruited with well-defined residential status. The red, brown and green coloured panels show the distribution of lineage 4, lineage 5 and lineage 6 respectively. All other sub-lineages/species have been indicated in the respective legends. This figure was created using the ArcMap program in ArcGIS v. 10.0. Abbreviations: MTBC, *Mycobacterium tuberculosis* complex; MTBss, *Mycobacterium tuberculosis* sensu stricto; Maf, *Mycobacterium africanum*; L4, Lineage 4; L5, Lineage 5; L6, Lineage 6; Ghana, Ghana genotypes (Ghana sub-lineage); Cam, Cameroon sub-lineage; MamE, Mamprusi East district; TamM, Tamale Metropolis; AgWM, Agona West Municipal; GomE, Gomoa East; AwuS, Awutu Senya; GaSM, Ga South Municipal; GaWM, Ga West Municipal; GaCM, Ga Central Municipal; GaEM, Ga East Municipal; AMA, Accra Metropolis; LaNM, La-Nkwantanang Madina Municipal; AdeM, Adenta Municipal; Kpes, Kpeshie Municipal.

Even though the sample size changed over time due to increase in case study sites, the proportion of distinct species did not change over time (Fig 3). The species/lineages/sub-lineages distributions of MTBC within the 13 administrative districts where participants resided are displayed in Fig 4.

### Spatial and space-time clustering analysis of MTBC cases at the district/sub-district level (2012–2014)

**Fig 5:**
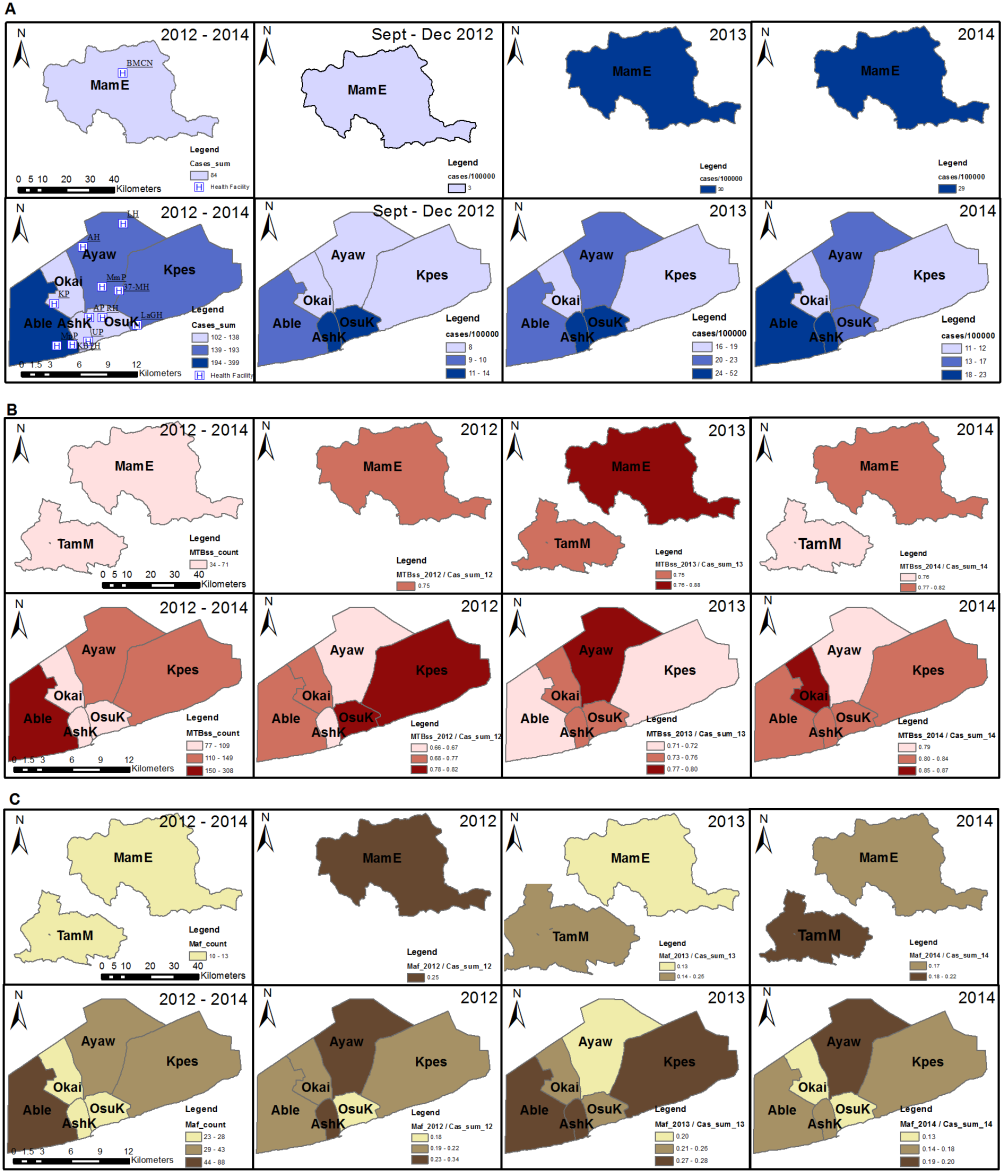
Spatial distribution of isolated MTBC within selected districts (2012–2014). This figure shows the; (A) Sum and case notification rate of all TB cases from September 2012 to December 2014, (B) Sum and normalized distribution of MTBss cases, (C) Sum and normalized distribution of Maf. The total number of cases per year was used as the denominator for normalization. Sampling from TamM did not meet our criteria for being included in analyses for case notification rate and so was excluded in all columns of panel A. Likewise we also recorded no cases in 2012 as such TamM was excluded from 2012 analysis (panel B and C). This figure was created using the ArcMap program in ArcGIS v. 10.0. Abbreviations: MTBss, *Mycobacterium tuberculosis* sensu stricto; Maf, *Mycobacterium africanum*; MamE, Mamprusi East district; TamM, Tamale Metropolis; AshK, Ashiedu Keteke; Ayaw, Ayawaso; Able, Ablekuma; OsuK, Osu Klottey; Okai, Okaikoi; Kpes, Kpeshie.

Spatial and space-time analyses were carried out for districts where sampling was performed within the time period; September 2012 to December 2014. These districts were AMA (sub-divided into sub-districts due to the population density) in the south and in the north, MamE. The TB case notification rate ranged from 3 to 52 cases/100,000 individuals at risk within the districts/sub-district analyzed (Fig 5A) with the highest case notification rate occurring in 2013 (52 cases/100,000). In a purely spatial analysis, we found two significant clusters within the study period based on cases notified. The most likely cluster consisted of two sub-districts, AshK and OsuK (p = 0.0000, RR = 3.99) with a secondary cluster occurring at MamE, (p = 0.0000, RR = 2.16) (Table 2). Similar observations were made using a space-time analysis with the likely clusters occurring in 2013. To analyze the spatial and space-time distribution of the two human adapted MTBC (MTBss and Maf), we normalized the relative district case frequencies to that for all cases obtained per district/year (Fig 5B and 5C). We found that the normalized distribution of both MTBss and Maf fluctuated over the three-year period, and no particular district/sub-district showed constant high values (Fig 5B and 5C). In a purely spatial analysis, significant clusters (p = 0.0000, Table 2) were observed in 6 of the 8 districts (MamE, TamM, Ayaw, Okai, Kpes and OsuK).

**Table 2:** Most likely spatial clusters detected in the study area using SaTScan analysis.

TB cases	Reference population	Cluster type	Year (s) of observed cluster	Clustered districts	Observed cases	Expected cases	Log Likelihood ratio	Relative risk	P-value	Type of analysis
All TB cases	District population	Most likely	2012–2014	AshK, OsuK	213	61.2	123.9	3.99	0.000	Purely spatial
All TB cases	District population	Secondary	2012–2014	TamM, MamE	138	67.9	29.8	2.16	0.000	Purely spatial
All TB cases	District population	Most likely	2013	AshK, OsuK	123	29.7	85.1	4.48	0.000	Space-time
All TB cases	District population	Secondary	2013	TamM, MamE	50	20.5	15.4	2.5	0.000	Space-time
MTBss	All cases per district	Most likely	2012–2014	MamE, TamM, Ayaw, Okai, Kpes, OsuK	588	398.1	75.0	2.21	0.000	Purely spatial
Maf	All cases per district	Most likely	2012–2014	MamE, TamM, Ayaw, Okai, Kpes, OsuK	158	107.8	18.9	2.12	0.000	Purely spatial
L4	All L4 cases in 2012	Most likely	2012–2014	Able	285	279.1	0.1	1.03	1.000	Purely spatial
L4	All L4 cases in 2012	Most likely	2012	Able	53	37.1	3.2	1.46	0.204	Space-time
Gh	All L4 cases in 2014	Most likely	2014	MamE, TamM	23	10.8	5.6	2.27	0.013	Space-time
Cam	All L4 cases in 2014	Most likely	2014	Kpes, Okai, Ayaw, TamM, MamE	122	80.0	11.5	1.68	0.000	Space-time
Cam	All L4 cases in 2013	Secondary	2013	AshK, OsuK	52	30.4	6.8	1.79	0.003	Space-time

This table shows the most likely spatial clusters detected from the SaTScan analysis. The TB case in the first column shows the category of TB lineage/sub-lineage to which the spatial or space-time analysis was performed. The districts to which the clusters were observed are shown in column 5 with the respective year of cluster observation shown in column 4. The last column shows the type of cluster analysis performed. *Abbreviations*: TB, tuberculosis; MTBss, *Mycobacterium tuberculosis* sensu stricto; Maf, *Mycobacterium africanum*; L4, Lineage 4; Gh, Ghana genotypes (Ghana sub-lineage); Cam, Cameroon sub-lineage; MamE, Mamprusi East; TamM, Tamale Metropolis; AshK, Ashiedu Keteke; Ayaw, Ayawaso; Able, Ablekuma; OsuK, Osu Klottey; Okai, Okaikoi; Kpes, Kpeshie.

**Fig 6:**
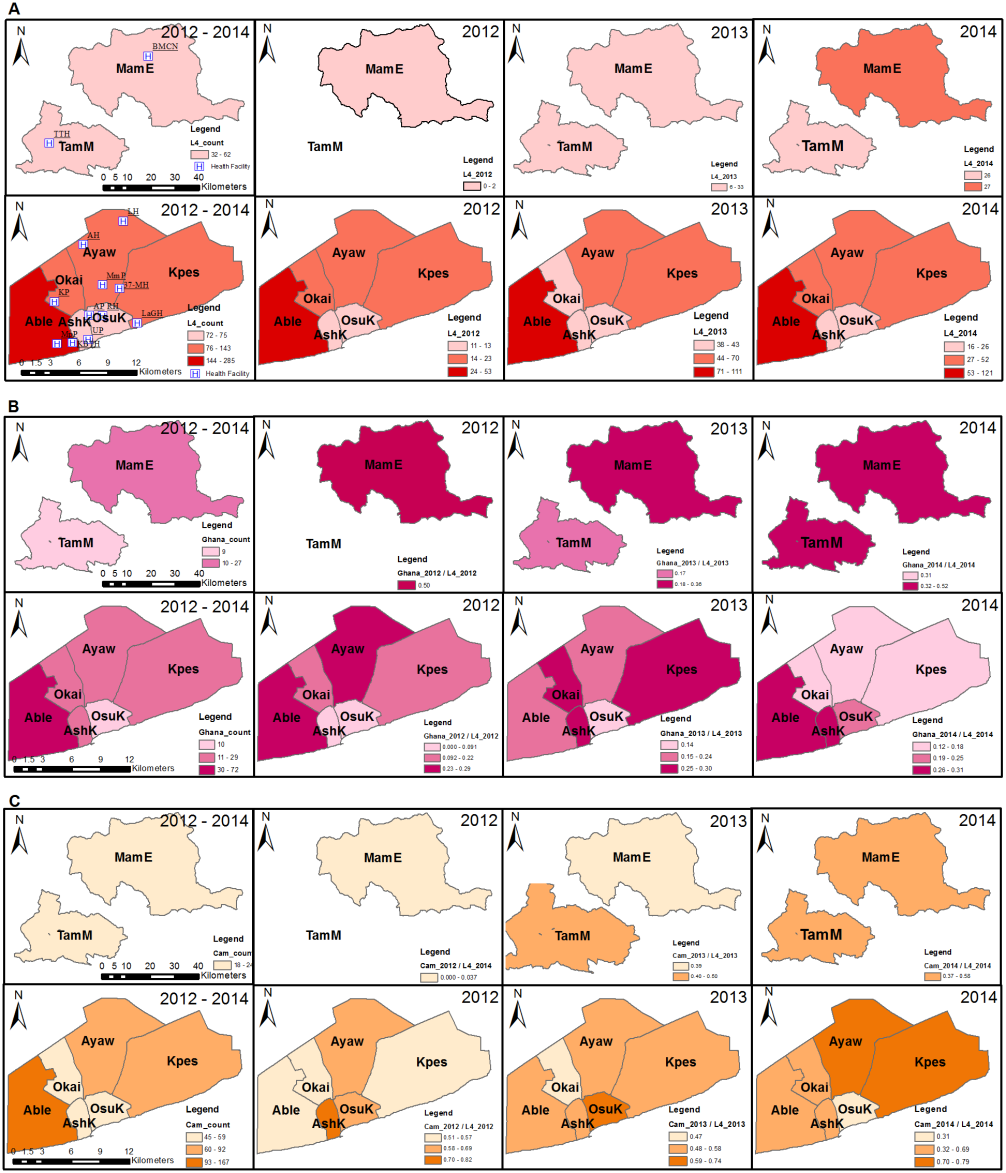
Spatial distribution of lineage 4 and major lineage 4 sub-lineages within selected districts (2012–2014). The figure shows the (A) Sum and normalized distribution of lineage 4 cases, (B) Sum and normalized distribution of Ghana sub-lineages cases, (C) Sum and normalized distribution of Cameroon sub-lineage cases. The total number of cases per year was used as the denominator for normalization. This study recorded no TB cases for TamM in 2012, consequently TamM was excluded from all analysis carried out using 2012 data. This figure was created using the ArcMap program in ArcGIS v. 10.0. Abbreviations: L4, Lineage 4; Ghana, Ghana genotypes (Ghana sub-lineage); Cam, Cameroon sub-lineage; MamE, Mamprusi East district; TamM, Tamale Metropolis; AshK, Ashiedu Keteke; Ayaw, Ayawaso; Able, Ablekuma; OsuK, Osu Klottey; Okai, Okaikoi; Kpes, Kpeshie.

The Ghana sub-lineage was found to significantly cluster in the north (p = 0.013, RR = 2.27). A space-time analysis revealed two significant clusters for the Cameroon sub-lineage with the most likely cluster occurring in 2014 (p = 0.000, RR = 1.68) consisting of five districts (Kpes, Okai, Ayaw, TamM and MamE). The second Cameroon sub-lineage cluster involved AshK and OsuK, which occurred in 2013 (p = 0.003, RR = 1.79, Fig 6C). Comparing the North and South for association with some risk factors showed association of the North with rural settings (P = 0.0000), farming (0.0000), contact with cattle (0.0003), compound housing (0.0000) whereas the South was associated with driving as occupation (0.0163) as shown in supplementary data (S6 Table).

## Discussion

Our objective was to analyse in time and space the prevalence of MTBC species and genotypes among isolates obtained from sputum-positive TB cases over an 8-year period in Ghana. A secondary objective was to determine the space clustering of specific genotypes. Our longitudinal analysis indicated that: 1) the Beijing and Ghana genotypes of Lineages 2 and 4, respectively, as well as the animal adapted MTBCs are isolated more often from patients from Northern than Southern Ghana and 2) the proportion of Maf among the isolates over the study period remained fairly constant.

Our first evidence of an association between the north and the Ghana genotype of Lineage 4 as well as the Beijing genotype of Lineage 2 reiterates the phylogeographical nature of the human-adapted MTBCs such that even within a single country there can be variations in the distribution of distinct genotypes within a lineage and specific geographical regions. For example in Senegal, it has been observed that the proportion of *M*. *africanum* causing TB varies by region [42].

The Ghana genotype clustered in the two districts (MamE and TamM) of the north (Fig 6B) whereas the Cameroon sub-lineage clustered in the South as two clusters (Fig 6C; Table 2). Thus clustering alone cannot be used as a proxy for active transmission of these genotypes in the specific geographical areas as the resolution of the molecular tool (spoligotyping) used for characterisation within this study is not enough to infer on-going transmission because of its low discriminatory power. However, they may be indicative of the areas of origin or introduction of these genotype/spoligotypes into the country. The Cameroon genotype as observed from various molecular studies is the most prevalent genotype causing TB in West Africa and our findings confirm this and also show that this genotype may have been introduced into the country through the south. Our findings call for studies to investigate the transmission dynamics of these sub-lineages within the respective geographical areas; this is of public health concern because evidence that MTBC genotypes might influence disease phenotype. For example, an association between Beijing strains (Lineage 2) and drug resistance has often been reported, and we recently showed that the Ghana genotype is also associated with drug resistance in Ghana (manuscript submitted). This means that effective control of TB in the Northern region of Ghana would be challenged with these two genotypes in higher proportions.

We also found the animal-adapted MTBC to be statistically associated with Northern Ghana (p = 0.0381, OR = 3.73). There were 5 patients among our study population who had direct contact with cattle including 4 butchers and 1 farmer who owns cattle and all 5 were infected with animal strains of the MTBC. This finding supports previous observations that people who are in direct constant contact with cattle and/or their products may be at risk of infection with *M*. *bovis* [43]. However, there were some patients from whom animal strains were isolated but did not have constant direct contact with cattle or any other farm animal. This finding compares with a similar work done in Mexico where most of the patients from whom animal strains of the MTBC were isolated had no direct contact with livestock but rather had consumed unpasteurized milk products in the past [44]. Our work design did not include analysis of patients’ dietary lifestyle and therefore we have no data on whether those patients are in a similar situation. Nevertheless, this association could be due to the dominance of livestock in the Northern region as compared to the south [26] meaning more possible interaction of humans with animals in the north as compared to the south.

The proportion of MTBss analysed among our data set remained stable and higher than that of Maf over the eight-year period (Figs 2A and 3). This result is in contrast with reports from other West African countries, indicating a decline of Maf [20, 21]. Various reasons have been suggested to explain the observed decline including non-specificity of biochemical assays used previously. Nevertheless, this study shows that Maf has remained fairly constant over the study period at an average of approximately 20% with significant fluctuations observed within four different periods {(2009/2010, P = 0.0422), (2010/2011, P = 0.0033), (2011/2012, P = 0.0002), (2013/2014, P = 0.0014)}.

This finding in conjunction with others [45–47] indicates that the two TB causing pathogens have adapted well within the Ghanaian population. Moreover, two independent studies conducted by our group found a strong association between L5 and an ethnic group in Ghana [[25], Asante-Poku *et al*, submitted].

### Study limitations

We did not collect GPS coordinate of the residence of the study participants, so each participant residential district was generated using his or her residential addresses from the questionnaire. This might not be accurate and so future studies should be done using GPS coordinates taken from each individual participant’s actual residence.

## Supporting Information

S1 Table Districts and population statistics within designated time points within the study period. The last population census conducted in Ghana was in 2010. As a results to obtain the population statistics for the period 2012 to 2014 (columns 5 to 7) we used the exponential growth rate formulae as described in methods. The intercensal growth rates used per region were: Greater Accra (3.1%), Central region (3.1%), Northern region (2.9%). *sub-districts within AMA; **†***Projected population from 2010 population census data*.
(PDF)

S2 Table Annual distribution of TB cases used for spatial or space-time analysis (2007–2014). The table lists the number of tuberculosis cases sampled within each district/sub-district for only participants with residential status. Periods within the 8 year study period where no sampling was done are marked N/A (not available). The final column and row contains total counts for each district/sub-district and year respectively. *sub-districts within AMA.
(PDF)

S3 Table Lineage distribution of isolated MTBC recruited and used for spatial or space-time analysis (2007–2014). The table lists the distribution of tuberculosis species/major lineages/sub-lineages sampled within each district/sub-district for only participants with well-defined residential status. The final column and row contains total counts for each district/sub-district and TB species/lineage/sub-lineage respectively. Abbreviations: TB, tuberculosis; MTBss, Mycobacterium tuberculosis sensu stricto; Maf, Mycobacterium africanum; L4, Lineage 4; L5, Lineage 5; L6, Lineage 6; Gh, Ghana genotypes (Ghana sub-lineage); Cam, Cameroon sub-lineage. *sub-districts within Accra metropolis.
(PDF)

S4 Table Default parameters used in SaTScan for clustering analysis. The table contains a list of the default settings used in performing the clustering analysis using the SaTScan software.
(PDF)

S5 Table Primer sequences for large sequence polymorphism (LSP) assay. The table contains a list of primer sequences used for the LSP assay.
(PDF)

S6 Table Comparison of some risk factors among the two regions. (PDF)

S7 Table Genotyping profile of 2551 MTBC isolates from Ghana. (PDF)

S8 Table Distribution of Species and Lineages of MTBC. (PDF)
