# Supplementary material for: Spatio-Temporal Distribution of Mycobacterium tuberculosis Complex Strains in Ghana
Source: PLoS One. 2016 Aug 26;11(8):e0161892. doi: 10.1371/journal.pone.0161892 (PMC5001706; doi:10.1371/journal.pone.0161892)
Supplement: S1 Table — The last population census conducted in Ghana was in 2010. As a results to obtain the population statistics for the period 2012 to 2014 (columns 5 to 7) we used the exponential growth rate formulae as described in methods. The intercensal growth rates used per region were: Greater Accra (3.1%), Central region (3.1%), Northern region (2.9%). *sub-districts within AMA; †Projected population from 2010 population census data. (PDF) [file pone.0161892.s001.pdf]

S1 Table. Districts and population statistics within designated time points within the study period

District/sub-district	Abbreviation	Region	2010	2012†	2013†	2014†
**Accra Metropolis**	AMA	Greater Accra	1665086	1771589	1827368	1884904
**Ablekuma***	Able	Greater Accra (AMA)	679362	722816	745574	769049
**Ashiedu Keteke***	AshK	Greater Accra (AMA)	117525	125042	128979	133040
**Ayawaso***	Ayaw	Greater Accra (AMA)	396487	421847	435129	448830
**Okaikoi***	Okai	Greater Accra (AMA)	349989	372375	384100	396193
**Osu Klottey***	OsuK	Greater Accra (AMA)	121723	129509	133586	137792
**Kpeshie**	Kpes	Greater Accra	411460	437778	451562	465779
**Adenta Municipal**	AdeM	Greater Accra	78215	83218	85838	88541
**La-Nkwantanang Madina Municipal**	LaNM	Greater Accra	111926	119085	122835	126702
**Ga Central Municipal**	GaCM	Greater Accra	117220	124718	128644	132695
**Ga East Municipal**	GaEM	Greater Accra	147742	157192	162141	167246
**Ga South Municipal**	GaSM	Greater Accra	411377	437690	451471	465685
**Ga West Municipal**	GaWM	Greater Accra	219788	233846	241209	248804
**Mamprusi East**	MamE	Northern	121009	128235	132008	135893
**Tamale Metropolis**	TamM	Northern	371351	393526	405106	417026
**Gomoa East**	GomE	Central	207071	220316	227252	234408
**Agona West Municipal**	AgWM	Central	115358	122737	126601	130587
**Ewutu Senya**	AwuS	Central	195306	207798	214341	221089

The last population census conducted in Ghana was in 2010. As a results, to obtain the population statistics for the period 2012 to 2014 (columns 5 to 7) we used the exponential growth rate formulae as described in methods. The intercensal growth rates used per region were: Greater Accra (3.1%), Central region (3.1%), Northern region (2.9%).

**sub-districts within AMA; †Projected population from 2010 population census data.*
