# Supplementary material for: Spatio-Temporal Distribution of Mycobacterium tuberculosis Complex Strains in Ghana
Source: PLoS One. 2016 Aug 26;11(8):e0161892. doi: 10.1371/journal.pone.0161892 (PMC5001706; doi:10.1371/journal.pone.0161892)
Supplement: S2 Table — The table lists the number of tuberculosis cases sampled within each district/sub-district for only participants with residential status. Periods within the 8 year study period where no sampling was done are marked N/A (not available). The final column and row contains total counts for each district/sub-district and year respectively. *sub-districts within AMA. (PDF) [file pone.0161892.s002.pdf]

S2 Table. Annual distribution of TB cases used for spatial or space-time analysis (2007 – 2014)

District/sub-district	2007	2008	2009	2010	2011	2012	2013	2014	Total per district
**Accra Metropolis**	N/A	N/A	N/A	119	141	N/A	N/A	N/A	260
**Ablekuma***	N/A	N/A	N/A	7	2	72	174	153	408
**Ashiedu Keteke***	N/A	N/A	N/A	5	N/A	18	53	31	107
**Ayawaso***	N/A	N/A	N/A	N/A	N/A	32	90	71	193
**Okaikoi***	N/A	N/A	N/A	N/A	N/A	30	62	46	138
**Osu Klottey***	N/A	N/A	N/A	1	N/A	17	70	24	112
**Kpeshie***	N/A	N/A	N/A	38	25	34	86	51	234
**Adenta Municipal**	N/A	N/A	N/A	N/A	N/A	4	3	3	10
**La-Nkwantanang Madina**	N/A	N/A	N/A	N/A	N/A	2	12	6	20
**Ga Central Municipal**	N/A	N/A	N/A	N/A	N/A	4	11	16	31
**Ga East Municipal**	N/A	N/A	N/A	N/A	N/A	15	23	11	49
**Ga South Municipal**	N/A	N/A	N/A	N/A	N/A	4	17	18	39
**Ga West Municipal**	N/A	N/A	N/A	1	N/A	6	19	11	37
**Mamprusi East**	N/A	N/A	N/A	N/A	N/A	7	42	44	93
**Tamale Metropolis**	N/A	N/A	N/A	N/A	N/A	N/A	8	37	45
**Agona West Municipal**	2	57	31	22	6	N/A	N/A	N/A	118
**Ewutu Senya**	8	59	18	17	N/A	7	35	21	165
**Gomoa East**	1	13	6	3	N/A	N/A	N/A	N/A	23
**Total per year**	11	129	55	213	174	252	705	543	2082

The table lists the number of tuberculosis cases sampled within each district/sub-district for only participants with residential status. Periods within the 8 year study period where no sampling was done are marked N/A (not available). The final column and row contains total counts for each district/sub-district and year respectively.

**sub-districts within AMA.*
