# Supplementary material for: Spatio-Temporal Distribution of Mycobacterium tuberculosis Complex Strains in Ghana
Source: PLoS One. 2016 Aug 26;11(8):e0161892. doi: 10.1371/journal.pone.0161892 (PMC5001706; doi:10.1371/journal.pone.0161892)
Supplement: S3 Table — The table lists the distribution of tuberculosis species/major lineages/sub-lineages sampled within each district/sub-district for only participants with well-defined residential status. The final column and row contains total counts for each district/sub-district and TB species/lineage/sub-lineage respectively. Abbreviations: TB, tuberculosis; MTBss, Mycobacterium tuberculosis sensu stricto; Maf, Mycobacterium africanum; L4, Lineage 4; L5, Lineage 5; L6, Lineage 6; Gh, Ghana genotypes (Ghana sub-lineage); Cam, Cameroon sub-lineage. *sub-districts within Accra metropolis. (PDF) [file pone.0161892.s003.pdf]

**S3 Table. Lineage distribution of isolated MTBC recruited and used for spatial or space-time analysis (2007 – 2014)**

| | TB Species/Lineages/Sub-lineages | | | | | | | | |
|---|---|---|---|---|---|---|---|---|---|
| **Districts/ sub-districts** | **MTBss** | **Maf** | **L4** | **L5** | **L6** | **Gh** | **Cam** | **Animal** | **Total** |
| **Accra Metropolis (2010/2011)** | 215 | 45 | 207 | 35 | 10 | 0 | 155 | - | 260 |
| **Ablekuma*** | 315 | 90 | 292 | 59 | 31 | 72 | 168 | 3 | 408 |
| **Ashiedu Keteke*** | 81 | 26 | 79 | 12 | 14 | 20 | 46 | - | 107 |
| **Ayawaso*** | 149 | 43 | 143 | 26 | 17 | 29 | 92 | 1 | 193 |
| **Okaikoi*** | 109 | 28 | 105 | 16 | 12 | 25 | 59 | 1 | 138 |
| **Osu Klottey*** | 87 | 24 | 72 | 15 | 9 | 10 | 46 | 1 | 112 |
| **Kpeshie*** | 182 | 51 | 168 | 43 | 8 | 27 | 101 | 1 | 234 |
| **Adenta Municipal** | 6 | 4 | 5 | 3 | 1 | 2 | 1 | - | 10 |
| **La-Nkwantanang Madina** | 17 | 3 | 17 | 2 | 1 | 3 | 13 | - | 20 |
| **Ga Central Municipal** | 27 | 4 | 27 | 2 | 2 | 3 | 18 | - | 31 |
| **Ga East Municipal** | 38 | 11 | 36 | 6 | 5 | 7 | 24 | - | 49 |
| **Ga South Municipal** | 31 | 8 | 29 | 6 | 2 | 8 | 17 | - | 39 |
| **Ga West Municipal** | 31 | 5 | 25 | 3 | 2 | 7 | 15 | 1 | 37 |
| **Agona West Municipal** | 105 | 13 | 102 | 10 | 3 | 0 | 63 | - | 118 |
| **Ewutu Senya** | 127 | 36 | 118 | 26 | 10 | 7 | 77 | 2 | 165 |
| **Gomoa East** | 18 | 5 | 15 | 3 | 2 | 0 | 9 | - | 23 |
| **Mamprusi East** | 79 | 13 | 69 | 7 | 6 | 28 | 27 | - | 92 |
| **Tamale Metropolis** | 34 | 10 | 32 | 4 | 6 | 9 | 18 | 1 | 45 |
| **Total** | 1651 | 420 | 1541 | 279 | 141 | 257 | 949 | 11 | 2082 |

The table lists the distribution of tuberculosis species/major lineages/sub-lineages sampled within each district/sub-district for only participants with well-defined residential status. The final column and row contains total counts for each district/sub-district and TB species/lineage/sub-lineage respectively.

Abbreviations: TB, tuberculosis; MTBss, Mycobacterium tuberculosis sensu stricto; Maf, Mycobacterium africanum; L4, Lineage 4; L5, Lineage 5; L6, Lineage 6; Gh, Ghana genotypes (Ghana sub-lineage); Cam, Cameroon sub-lineage.

**sub-districts within Accra metropolis.*
