# Supplementary material for: Spatio-Temporal Distribution of Mycobacterium tuberculosis Complex Strains in Ghana
Source: PLoS One. 2016 Aug 26;11(8):e0161892. doi: 10.1371/journal.pone.0161892 (PMC5001706; doi:10.1371/journal.pone.0161892)
Supplement: S4 Table — The table contains a list of the default settings used in performing the clustering analysis using the SaTScan software. (PDF) [file pone.0161892.s004.pdf]

S4 Table. Default parameters used in SaTScan for clustering analysis

Category	Parameters	Settings
**Input**	Time precision	Year
	Coordinates	Latitude/Longitude
**Data checking**	Temporal data check	Check to ensure that all cases and controls are within the specified temporal study period
	Geographical data check	Check to ensure that all observations (cases, controls and populations) are within the specified geographical area
**Analysis**	Time aggregation units	Year
	Time aggregation length	1
**Spatial window**	Maximum spatial cluster size	50% of population at risk
**Temporal window**	Minimum temporal cluster size	1 year
	Maximum temporal cluster size	50% of study period
**Inference**	Number of replications	999

The table contains a list of the default settings used in performing the clustering analysis using the SaTScan software.
