# Supplementary material for: Spatio-Temporal Distribution of Mycobacterium tuberculosis Complex Strains in Ghana
Source: PLoS One. 2016 Aug 26;11(8):e0161892. doi: 10.1371/journal.pone.0161892 (PMC5001706; doi:10.1371/journal.pone.0161892)
Supplement: S5 Table — The table contains a list of primer sequences used for the LSP assay. (PDF) [file pone.0161892.s005.pdf]

S5 Table. Primer Sequence for large sequence polymorphism LSP Assays

Region of Difference	Sequence (5’-3’)
**RD4-Flanking Forward**	CTC GTC GAA GGC CAC TAA AG
**RD4-Flanking Reverse**	AAG GCG AAC AGA TTC AGC AT
**RD9-Flanking Forward**	ACT CCC AGC GCT CGG CGG TGA CGG TAT CGT
**RD9-Flanking Reverse**	ATT CCG TGG GCG CTG CGG CCA ATG TTT GTT
**RD12-Flanking Forward**	GCC ATC AAC GTC AAG AAC CT
**RD12-Flanking Reverse**	CGG CCA GGT AAC AAG GAG T
**RD702-Flanking Forward**	TTC CGA GGA CCC GTT GTT GAG TGC
**RD702-Flanking Reverse**	GGG CGG GTT GGG TTG CTG GTC
**RD711-Flanking Forward**	GGC CGC CCT GCT CAA GAA CCT
**RD711-Flanking Reverse**	CCT AGG CCG GCG ACG AAG TG

The table contains a list of primer sequences used for the LSP assay
