# Supplementary material for: Spatio-Temporal Distribution of Mycobacterium tuberculosis Complex Strains in Ghana
Source: PLoS One. 2016 Aug 26;11(8):e0161892. doi: 10.1371/journal.pone.0161892 (PMC5001706; doi:10.1371/journal.pone.0161892)
Supplement: S6 Table — (PDF) [file pone.0161892.s006.pdf]

S6 Table. Comparison of some risk factors among the two regions

Variables	North	South	P-value	OR	95% CI
Settlement (1938)	***212***	***1726***			
Rural (152)	151 (71.2%)	1 (0.1%)	0.0000	4154.6	$6.9e^{+2}$-$4.5e^{+15}$
Urban (1786)	61(28.8%)	1725 (99.9%)	0.0000	0.0	0.0-$1.4e^{-3}$
Occupation (1468)	***168***	***1300***			
Farmer (108)	81 (48.2%)	27 (2.1%)	0.0000	43.6	26.4-74.2
Driver (156)	9 (5.4%)	147 (11.3%)	0.0163	0.4	0.2-0.9
Constant contact with Cattle (7) *	5 (3.0%)	2 (0.2%)	0.0003	19.8	3.2-209.3
Housing (1459)	***169***	***1290***			
Compound (1207)	160 (96.7%)	1047 (81.2%)	0.0000	4.1	2.1-9.3
Self-Contained (252)	9 (3.3%)	243 (18.8%)	0.0000	0.2	0.1-0.5
HIV (732)	***50***	***682***			
Positive (102)	3 (6.0%)	99 (14.5%)	0.1350	0.4	0.1-1.2
Negative (630)	47 (94.0%)	583 (85.5%)	0.1350	2.7	0.8-13.6

*there were 5 butchers (Abattoir workers) and 2 farmers who own cattle
