# Supplementary material for: Spatio-Temporal Distribution of Mycobacterium tuberculosis Complex Strains in Ghana
Source: PLoS One. 2016 Aug 26;11(8):e0161892. doi: 10.1371/journal.pone.0161892 (PMC5001706; doi:10.1371/journal.pone.0161892)
Supplement: S7 Table — (PDF) [file pone.0161892.s007.pdf]

Supplementary table 7: Genotyping profile of 2551 MTBC isolates from Ghana

Specie	SNP	Spoligotyping profile	Sublineage	SIT	No	%
MTBss	L1	■■■■■■■■■■■■■■■■■■■■■■■■■■■■□□□□■□■■■■■■■■■	EAI	236	6	0.20
MTBss	L1	■□□■■■■□□□■■■■■■■■■■■■■■■■■■□□□□■□■■■■■■■■■	EAI	340	11	0.40
MTBss	L1	■■□■■■■■■■■■■■■■■■■■■■■■■■■■□□□□■□■■■■■■■■■	EAI	342	15	0.60
MTBss	L1	■□■■■■■■■■■■■■■■■■■■■■■■■■■■□□□□■□■■■■■■■■■	EAI	380	2	0.07
MTBss	L1	■■□■■■■■■■■■■■■■■■□□■□□■■■■■□□□□■□■■■■■■■■■	EAI	Orphan	1	0.04
MTBss	L1	■□■■■■□□□□□■■■■■■■■■■■■■■■■■□□□□■□■■■■■■■■■	EAI	Orphan	1	0.04
MTBss	L2	□□□□□□□□□□□□□□□□□□□□□□□□□□□□□□□□□□■■■■■■■■■	Beijing	1	71	3.00
MTBss	L2	□□□□□□□□□□□□□□□□■□□□□□□□□□□□□□□□□□■■■■■■■■■	Beijing	1184	3	0.12
MTBss	L2	□□□□□□□□□□□□□□□□□□□□□□□□□□□□□□□□□□■■■■■■□■■	Beijing	941	2	0.07
MTBss	L2	□□□□□□□□□□□□□□□□□□□□□□□□□□□□□□□□□□■■■■■■■■□	Beijing		1	0.04
MTBss	L3	■■■□□□□■■■■■■■■■■■■■■■□□□□□□□□□□□□■■□□■■■■■	Dehli_CAS	25	4	0.20
MTBss	L3	■■■□□□□□□■■■■■■■■■■■■■□□■■■■□□□□■□■■■■■■■■■	Dehli_CAS	129	2	0.07
MTBss	L3	■■■□□□□□□■■■■■■■□■■■■■□□■■■■□□□□■□■■■■■■■■■	Dehli_CAS	702	3	0.12
MTBss	L3	■■■□□□□■■■■■■■■■■■■■■■□□□□□□□□□□□□□■□□■■■■■	Dehli_CAS	1199	4	0.20
MTBss	L3	■■■□□□□■■■□□■■■■■■■■■■□□■□□□□□□■■■□□□□□□□□□	Dehli_CAS		2	0.07
MTBss	L3	■■■□□□□■■■■■■■■■■■■■■■□□■□□□□□□■■■■■■■■■■■■	Dehli_CAS		3	0.12
MTBss	L3	■■■■□□□■■■■■■■■■■■■■■■□□□□□□□□□□□□■■■■■■■■■	Dehli_CAS		2	0.07
MTBss	L3	■■■□□□□□■□■■■■■■■■■■■■□□■□□□□□□■■■■■■■■■■■■	Dehli_CAS		3	0.12
MTBss	L4	■■■■■■■■■■■■■■■■■■□□■■□□□■■■■■■■□□□□■■■■■■■	Cameroon	57	13	0.50
MTBss	L4	■■■■■■■■■■■■■■■■■■■■■■□□□■■■■■■■□□□□■■■■■■■	Cameroon	61	811	32.00
MTBss	L4	■■■■■■■■■■■■■■□■■■■■■■□□□□■■■■■■□□□□■■■■■■■	Cameroon	114	1	0.04
MTBss	L4	■■■■■■■■■■■■■■■■■■■■■■□□□■■■■■■■□□□□■■■□■■■	Cameroon	403	5	0.20
MTBss	L4	■■■■■■■■■■■■■■■■■■■■■■□□□■■■□□■■□□□□■■■■■■■	Cameroon	772	92	3.60
MTBss	L4	■■■■■■■■■■■■■■■■■■■■■■□□□■■■■■■■□□□□■■■■□■■	Cameroon	838	28	1.10
MTBss	L4	■■■■■■■■■■■■■■■■■■■■■■□□□□■■■■■■□□□□■■■■■■■	Cameroon	1141	14	0.50
MTBss	L4	■■■■■■■■■■■■■■■■■■■■■■□□■■■■■■■■□□□□■■■■■■■	Cameroon	1580	15	0.60
MTBss	L4	■■■■■■■■■■■■■■■■■■■■■■□□□■■■□■■■□□□□■■■■■■■	Cameroon		25	1.00
MTBss	L4	■■■■■■■■■■■■■■□■■■■■■□□□□■■■■■■■□□□□■■□■■□■	Cameroon		35	1.40
MTBss	L4	■■■□□□□□□□□□□□□□□□□□□□□□□□□□□□□□□□□□■■■■■■	Cameroon		10	0.40
MTBss	L4	■■■■■■■■■■■■■■■■■■■■■■□□□□□□■■■■□□□□■■■■■■■	Cameroon		30	1.20
MTBss	L4	■■■■□■■■■■■■■■■■■■■■■■□□□■■■□□■■□□□□■■■■■■■	Cameroon		20	1.00
MTBss	L4	■■■■■■■■■■■■□■■■■■■■■■□□□□□□■■■■□□□□■■■■■■■	Cameroon		25	1.00
MTBss	L4	■■■■■■■■■■■■□■■■■■■■■■□□□■■■□□■■□□□□■■■■■■■	Cameroon		9	0.40
MTBss	L4	■■■■■■■■■■■■□■□■■■■■■■□□□■■■■■■■□□□□■■■■■■■	Cameroon		18	0.70
MTBss	L4	■■■■■■■■■■■■□■■■■■■■■■■■■■■■■■■■□□□□■■■■■■■	Ghana	37	2	0.07
MTBss	L4	■■■■■■■■■■■■■■■■■■■■■■□■■■■■■■■■□□□□■■■■■■■	Ghana	44	2	0.07
MTBss	L4	■■■■■■■■■■■■■■■■■■■■■■■■■■■■■■■■□□□□■■■■■■■	Ghana	53	269	11.0
MTBss	L4	■■■■■■■■■■■■■■■■■■■■■■■■■■■■■■■■□□■■■■■■■■■	Ghana	54	3	0.12
MTBss	L4	■■■■■■■■■■■■■■■■■■■□■■□■■■■■■■■■□□□□■■■■■■■	Ghana	58	1	0.04

Specie	SNP	Spoligotyping profile	Sub lineage	SIT	No	%
MTBss	L4	■■■■■■■■■■■■■■■■■■■■■■■■■■■■■■■■□□□□■■■■■□■	Ghana	278	1	0.04
MTBss	L4	■■■■■■■■■■■■■■■■■■■■■■■□■■■■■■■■□□□□■■■■■■■	Ghana	373	1	0.04
MTBss	L4	■■■■■■■■■■■■■■■■■■■■■■■■■■■■□■■■□□□□■■■■■■■	Ghana	462	3	0.12
MTBss	L4	■■■■■■■■■■■■□■■■■■■■■■■■□■■■■■■■■■■□□□□■■■■■■■	Ghana	504	28	1.10
MTBss	L4	■□□■■■■■■■■■■■■■■■■■■■■■■■■■■■■■□□□□■■■■■■■	Ghana	804	1	0.04
MTBss	L4	■■■■■■□■■■■■■■■■■■■■■■■■■■■■■■■■□□□□■■■■■■■	Ghana	926	1	0.04
MTBss	L4	■■■■■■■■■■■■■■■□■■■■■■■■■■■■■■■■□□□□■■■■■■■	Ghana	1105	1	0.04
MTBss	L4	■■■■■■■■■■■■■■■■■■■■■■■■■■■■■■■■□□□■■■■■■■■	Ghana	1196	3	0.12
MTBss	L4	■■■■■■■■■■■■□■■■■■■□■■□■■■■■■■■■□□□□■■■■■■■	Ghana	1227	3	0.12
MTBss	L4	■■■■■■■■■■■■□■□■■■■■■■■■■■■■■■■■□□□□■■■■■■■	Ghana	1547	1	0.04
MTBss	L4	■■■■■■■■■■■■■■■■■■■■■■■■■■■■■■■■□□□□■■□■■□■	Ghana		7	0.30
MTBss	L4	■■■■■■■■■□■■■□□■■■■■■■■■■■■■■■■■□□■■■■□■■■■	Ghana		3	0.12
MTBss	L4	■■■■■■■■■■■■□■■■■■■■■■■■□■■■■□■■■■■□□□□■■■■■■■	Ghana	orphan	1	0.04
MTBss	L4	■■■■■■■■■■■■■■■■■■■□□■■■■■■■■■■■□□□□■■■■■■■	H37Rv		2	0.07
MTBss	L4	■■■■■■■■■■■■□■■■■■■■■■■■■■■■■■□■□□□□■■■■■■■	Haarlem	36	1	0.04
MTBss	L4	■■■■■■■■■■■■■■■■■■■■■■■□■□□□□□□■□□□□■■■■■■■	Haarlem	45	3	0.12
MTBss	L4	■■■■■■■■■■■■■■■■■■■■■■■■■□□□□□□■□□□□■■■■■■■	Haarlem	47	1	0.04
MTBss	L4	■■■■■■■■■■■■■■■■■■■■■■■■■■■■■■□■□□□□■■■■■■■	Haarlem	50	51	2.00
MTBss	L4	■■■■■■■■■■■■■■■■■■■■■■■■■□□□□□□■□□□□■■■□■■■	Haarlem	62	1	0.07
MTBss	L4	■■■■■■■■■■■■■■■■■■■■■■■■■■■■■■□□□□□□■■■■■■■	Haarlem	124	1	0.04
MTBss	L4	■■■■■■□□□□□□□□□□□□□□□□□□□■■■■■■■□□□□■■■■■■■	Haarlem	144	2	0.04
MTBss	L4	■■■■■■■■■■■■■■■■■■■■■■■■□□□□□□□■□□□□■■■□■■■	Haarlem	316	8	0.30
MTBss	L4	■□□■■■■■■■■■■■■■■■■■■■■■■■■■■□■□□□□■■■■■■■	Haarlem	655	18	0.70
MTBss	L4	■■■■■■■■■■■■■■■■■■■■■■■■■■■□□□□□□□□□□■■■■■■	Haarlem	775	3	0.12
MTBss	L4	■□□□■■■■■■■■■■■■■■■■■■■■■■■■■■□■□□□□■■■■■■■	Haarlem	1159	1	0.04
MTBss	L4	■■■■■■■■■■■■■■■■■■■■■■■■■■□□□□□□□□□□□■■■■■■	Haarlem	1498	16	1.00
MTBss	L4	■■■■■■■■■■■■■■□■■■■■■■■■■■□□□□□□□□□□■■□■■□■	Haarlem		4	0.20
MTBss	L4	■■■■■■■■□■■■■■■■■■■■□□□□■■□□□□□■□□□□■■■■■■■	Haarlem		3	0.12
MTBss	L4	■■■■■□□□□□□□□□□□■□□□□□□□□□■■■■□■□□□□■■■□■■■	Haarlem		3	0.12
MTBss	L4	■■■■■■■■■■■■■■■■■■■■■■■■■■■■■■□■□□□□■■□■■□■	Haarlem		3	0.12
MTBss	L4	■■□■■■■■■■■■■■■■■■■■□□□□■■■■■■■■□□□□■■■■■■■	LAM	20	1	0.04
MTBss	L4	■■■■■■■■■■■■■■■■■■■■□□□□■■■■■■■■□□□□■■■■■■■	LAM	42	26	1.00
MTBss	L4	■■■■■■■■■■■■■■■■■■■■■□□□■■■■■■■■□□□□■■■■■■■	LAM	535	2	0.07
MTBss	L4	■■■■■■■■■■■■■■□□□□□□□■■■■■■■■■■■□□□□■■■■■■■	LAM	765	1	0.04
MTBss	L4	■■■■■■■■■■■■■■□□□■□□□■■■■■■■■■■■□□□□■■■■■■■	LAM		2	0.07
MTBss	L4	■■□■■■■□□□□□□□■■■■■■□□□□□■■■■■■■□□□□■■■■■■■	LAM	orphan	1	0.04
MTBss	L4	■■□■□■■■■■■□□■■■■□□■□□□□■■■■■■■■□□□□■■■■■■■	LAM		2	0.07
MTBss	L4	■■■■■■■■■■■■■□□□■■□□□□□■■■■■■■■■□□□□■■■■■■■	LAM		2	0.07
MTBss	L4	■■■■■■■■■■■■■■■■■■■■■■■■■■■■□□□□□□□□□■■■■■■	NEW	orphan	1	0.07
MTBss	L4	■■■■□□□□□□□■■■■■■□□■■■■■■■■■■■■■□□□□■■■■■■■	S		3	0.12

MTBss	L4	■■■■■■■■■■■■■■■■■■■■■■■■■■■■■■□■□□□□■■■□■■■	Uganda I	49	2	0.07
MTBss	L4	■■■■■■■■■■■■■■■■■■■■■■■■■■■■■■■■□□□□■■■□■■■	Uganda I	52	2	0.07
MTBss	L4	■■■■■■■■■■■■■■■■■■■■■■■■■■■■■■■■□□□□■■□□■□■	Uganda I	78	1	0.07
**Specie**	**SNP**	**Spoligotyping profile**	**Sublineage**	**SIT**	**No**	**%**
MTBss	L4	■■■□■■■■■■■■■■■■■■■■■■■■■■■■■■■■□□□□■■■□■■■	Uganda I	848	1	0.04
MTBss	L4	■■■■■■■■■■■■■■■■■■■■■■■■■■■■■■□■□□□□■■■□□■■	Uganda I	524	1	0.04
MTBss	L4	■■■■□□■■■■■■■■■■■■■■■■■■■■■■■■■■□□□□■■■□■■■	Uganda I	712	1	0.04
MTBss	L4	■■■■■■■■■■□□■■■■■■■■■■■■■■■■■■■■□□□□■■■□■■■	Uganda I	1056	1	0.04
MTBss	L4	■□□■■■■■■■■■■■■■■■■■■■■■■■■■■□■□□□□■■■□□■■	Uganda I		22	1.00
MTBss	L4	■■■■■■■■■■■■■■■■■■■■■■■■■■■■■■■■□□□□■■■□□■■	Uganda I		3	0.10
MTBss	L4	■■■■■■■■■■■■■■■■■■■■■■■■■■■■■■□■□□□□■■□□□□■	Uganda I		4	0.20
MTBss	L4	■■■■■■■■■■■■■■■■■■■■■■■■■■■■■■□□□□□□□□□□□□□	Uganda II	237	2	0.07
MTBss	L4	■■■□□□□□□□□□■■■■■□■■■■■■■■■■■■■■□□□□■■■■■■■	Uganda II	92	11	0.40
MTBss	L4	■■■□□□□□□□□□■■■■■□■■■■■■■■■■■■■■□□□□■■■□□□□	Uganda II	200	5	0.20
MTBss	L4	■■■■■■■■■■■■■■■□□■□□□□□□□□□□□□□□□□□□■■■□■■■	Uganda II	1178	4	0.20
MTBss	L4	■■□■■■■□□□□□■□■■■■■■□□□□■■■■■■■■□□□□■■■■■■■	Uganda II		5	0.20
MTBss	L4	■■■■■■■■□□□□■□■■■■■■■■■■■■■■■■■■□□□□■■■■■■■	Uganda II	orphan	1	0.04
MTBss	L4	■□□■■■■■■■■■■■□■■■■■■■■■■■■■■■□■□□□□■■■■■■■	Uganda II		6	0.20
MTBss	L4	■■■□□□□□□□□□■■■■■□□■■■■■■■■■■■■■□□□□■■■■■■■	Uganda II		2	0.07
MTBss	L4	■■□□□□■■■■■■■■■■■■■■■■■■■■□□□□□□□□□□□□■■■■■	Uganda II		4	0.20
MTBss	L4	■■■□□□□□□□□□■■■■■□□□□■■■■■■■■■■■□□□□■■■□□□□	Uganda II	orphan	1	0.04
MTBss	L4	■■■□□□□□□□□□■■■■■■■■■■■■■■■■■■■■□□□□■■■□□□□	Uganda II		3	0.10
MTBss	L4	■■■□□□□□□□□□■■■■■□□■■■■■■■■■■■■■□□□□■■■■■■■	Uganda II	orphan	1	0.04
Mafric	L5	■□■■■■■□□□□□■■■■■■■■□□□□■■■■■■■■■■■■□□□■■■■	West Africa I	319	52	2.00
Mafric	L5	■■■■■■□□□□□□□□□□■■■■□□□□■■□□■■■■■■■■□□□■■■■	West Africa I	320	10	0.40
Mafric	L5	■■■■■■■□□□□□■■■■■■■■□□□□■■■■■■■■■■■■□□□□■■■	West Africa I	330	11	0.40
Mafric	L5	■■■■■■■□□□□□■■■■■■■■□□□□■■■■■■■■■■■■□□□■■■■	West Africa I	331	61	2.40
Mafric	L5	■■■■■■■□□□□□■■■■■■■■■■■■■■■■■■■■■■■■□□□■■■■	West Africa I	438	14	1.00
Mafric	L5	■■■■■■■□□□□□■■■■■■■■□□□□□■□□□□□□□□□□□□□□■■■	West Africa I	1592	2	0.07
Mafric	L5	■□■■■■■□□□□□■■■■■■■■□□□□■■■■■■■■■■■■□□□■■■■	West Africa I		50	1.96
Mafric	L5	■■□■■■■□□□□□■■■■■■■■□□□□■■■■■■■■■■■■□□□■■■■	West Africa I		10	0.40
Mafric	L5	■■□■■■■□□□□□■■■■■■■■■□□■■■■■■■■■■■■■□□□■■■■	West Africa I		11	0.40
Mafric	L5	■■■■■■■□□□□□□■■□■■■■□□□□■■■■■■■■■■■■□□□■■■■	West Africa I		11	0.40
Mafric	L5	■□□■■■■□□□□□■■■■■■■■□□□□■■■■■■■■■■■■□□□■■■■	West Africa I		11	0.40
Mafric	L5	■■■■■■■□□□□□□■■■■■■■□□□□■■■■■■■■■■■■□□□■■■■	West Africa I		30	1.20
Mafric	L5	■■■■■■■□□□□□■■■■■□□□□□□□■■■■■■■■■■■■□□□■■■■	West Africa I		1	0.04
Mafric	L5	■■□■■■■□□□□□■■■■■■■■□□□□■■■■■■■■■■■■□□□□■■■	West Africa I		2	0.07
Mafric	L5	■■■■■■■□□□□□■■■■■■■□□□□□□□□■■□□□□□■■□□□□■□■	West Africa I		2	0.07
Mafric	L5	■■■■■■■□□□□□■■■■□□□□□□□□■■□□□□□■■■■■□□□■■■■	West Africa I		1	0.04

Specie	SNP	Spoligotyping profile	Sub lineage	SIT	No	
Mafric	L5	■■■□■■■□□□□□□■■■■■■■□□□□■■■■■■■■■■■■□□□■■■■	West Africa I	orphan	1	0.04
Mafric	L5	■■■■■■■□□□□□■■■■■■■□□□□□□□□■■■□□□■■■□□□□■■■	West Africa I	orphan	1	0.04
Mafric	L5	■■■■■■■□□□□□■■■■■■■■□□□□□□□□□□□□□□□□□□□□■■■	West Africa I		2	0.07
Mafric	L5	■■■■■■■□□□□□■■■■■■■□□□□□□■□■■■□□□■■■□□□□■■■	West Africa I	orphan	1	0.04
Mafric	L5	■□■■■■■□□□□□■■■■■■■■□□□□□□□□□□□□□□□□□□□□■■■	West Africa I		1	0.04
Mafric	L5	■■■■■■■□□□□■■■■■■■■■□□□□□■■□□□□□□□□□□□□□■■■	West Africa I	orphan	1	0.04
Mafric	L5	□□■■■■■□□□□□■■■■■■■■□□□□■■■□□■■■■■■■□□□■■■■	West Africa I	orphan	1	0.04
Mafric	L5	■■■■■□■□□□□□■■■■■■■■□□□□■■■■■■■■■■■■□□□■■■■	West Africa I		3	0.12
Mafric	L5	■■■■■■■□□□□□■■■■■□□□□□□□■■■■■■■■■■■■□□■■■■■	West Africa I	orphan	1	0.04
Mafric	L5	■□■■■■■□□□□□■■■■■■■■□□□□■■■■■■■■■□■■□□□■■■■	West Africa I	orphan	1	0.04
Mafric	L5	■■■■■■■□□□□□■■■■■□□□□□□□■■■■■■■■■■■■□□□■■■■	West Africa I		4	0.20
Mafric	L5	■■■■■■■□□□□□■■■■■■■□□□□□□■□■■□□□□□■■□□□□■■□	West Africa I		2	0.07
Mafric	L5	■■■■■■■□□□□□■■■■■■■■□□□□■■■■■■■■■■■■□□■■■■■	West Africa I		2	0.07
Mafric	L5	■■■■■■■□□□□□■■■■□□□□□□□□■■□□□□□■■■■■□□□■■■■	West Africa I		2	0.07
Mafric	L5	■■■■■■■□□□□□■■■■■■■■□□□□■■■■■■■□■■■■□□□■■■■	West Africa I		1	0.04
Mafric	L5	■■■■■■■□□□□■■■■■■■■□□□□□□■■■■□□□□□■■■■□□■■■	West Africa I	orphan	1	0.04
Mafric	L5	■■□■■■■□□□□□■■■■■■■■■□□■■■■■■■■■■■■■□□□■■■■	West Africa I	orphan	1	0.04
Mafric	L5	■■■■■■■□□□□□■■■■■■■□□□□■■■■■■■■■■■■■□□□■■■■	West Africa I		5	0.20
Mafric	L5	■■■■■■□□□□□□■■■■■■■■□□□□■■■■■■■■■■■■□□□■■■■	West Africa I		2	0.07
Mafric	L5	■■■■■■■□□□□□■■■■■■■■■□□□■■■■■■■■■■■■■□□□■■■	West Africa I	orphan	1	0.04
Mafric	L5	■□■□■■■□□□□□□■■■■■■■□□□□■■■■■■■■■■■■□□□■■■■	West Africa I	orphan	1	0.04
Mafric	L5	■■■■■■■□□□□□■■■■■■■■□□□□□□□□□□□□□□□□□□□□□□□	West Africa I		2	0.07
Mafric	L5	■■■■■■□□□□□□□□□□□□□□□□□■■■■■■■■■■■■■□□□■■■■	West Africa I	orphan	1	0.04
Mafric	L5	■■■■■■■□□□□□■■■■□□□□□□□□■■□□□□□■■■■■□□■■■■■	West Africa I	orphan	1	0.04
Mafric	L5	■■■■■■■□□□□□□■□■■■■■□□□□■■■■■■■■■■■■□□□■■■■	West Africa I	orphan	1	0.04
Mafric	L5	■■■■■■■□□□□□■■■■■■■■□□□□■■■■■■■□■■■■□□□■■■■	West Africa I	orphan	1	0.04
Mafric	L5	■□■■■■■□□□□□■■■■■■■■■□□□■■■■■■■■■■■■□□□■■■■	West Africa I		2	0.07

Specie	SNP	Spoligotyping profile	Sub lineage	SIT	No	
Mafric	L5	■■■■■■□□□■■■■■■■■■■■■□□□□■■□□□□□□□□□■□□■■■■	West Africa I	orphan	1	0.04
Mafric	L5	■■□■■■■□□□□□□■■■■■■■□□□□■■■■■■■■■■■■□□□□■■■	West Africa I		2	0.07
Mafric	L5	■■□■■■■□□□□□■■■■■■■■■□□■■■■■■■■■■■■■□□□■■■■	West Africa I	orphan	1	0.04
Mafric	L5	■□■■■■■□□□□□■■■■■■■□□□□■■■■■■■■■■■■■□□□■■■■	West Africa I		4	0.20
Mafric	L5	■■■■■■■□□□□□■■■■■□□□□□□□■■□□□□□■■■■■□□□■■■■	West Africa I	orphan	1	0.04
Mafric	L5	■■■■■■□□□□□■□■■□■■■□□□□□□□□□□□□□□□■■□□□■■■■	West Africa I	orphan	1	0.04
Mafric	L5	■■■■■■■□□□□□■■■■□□□□□□□□■■□□□□□■■■■■□□□■■■■	West Africa I	orphan	1	0.04
Mafric	L5	■■■■■■■□□□□□□■■■■■■■□□□□■■■■■■■■■■■■□□□■■■■	West Africa I	orphan	1	0.04
Mafric	L5	■□■■■■■□□□□□■□■■■■■■□□□□■■■■■■■■■■■■□□□■■■■	West Africa I	orphan	1	0.04
Mafric	L5	■□■■■■■□□□□□■■■■■■■■□□□□■■■□□■■■■■■■□□□■■■■	West Africa I	orphan	1	0.04
Mafric	L5	■■■■■■■□□□□□■■■■□□□□□□□□□□□□□□□■■■■■□□□■■■■	West Africa I	orphan	1	0.04
Mafric	L5	■■■■■■□□□□□□■■■■■■■■■□□□■■■■■■■■■■■■□□□■■■■	West Africa I	orphan	1	0.04
Mafric	L6	■■■■■■□□□■■■■■■■■■■■■■■■■■■■■■■■■■■■■□■■■■	West Africa II	181	55	2.20
Mafric	L6	■■■■■■□□□■■■■■■■■■■■■□□□■■■■■■■■■■■■■■□■■■■	West Africa II	326	40	2.00
Mafric	L6	■■■□□□□■■■■■■■■■■■■■■■□□■■■■■■■■■■■■■■■■■■■	West Africa II	1200	1	0.04
Mafric	L6	■■■■■■□□□■■■■■■■■■■■■□□□■■■■■■■■■■■■■■□■□■■	West Africa II	1867	1	0.04
Mafric	L6	■■■■■■□□□■■■■■■■■■■■■□□□■■■■■■■■■■■■■■□□■■■	West Africa II		4	0.20
Mafric	L6	■■■■■■□□□■■■□□■■■■■■■■■■■■■■■■■■■■■■■■□■■■■	West Africa II		4	0.20
Mafric	L6	■■■■■■■■■□■■■□□■■■■■■■■■■■■■■■■■■■■■■□■■■■	West Africa II	orphan	1	0.04
Mafric	L6	■■■■■■■■■□■■■■■■■■■■■■■■■■■■■■■■■■■■■■■■■	West Africa II	orphan	1	0.04
Mafric	L6	■■■■■■■□■□□□■□□■■■■■■■■■■■■■■■■■■■■■■□■■■■	West Africa II	orphan	1	0.04
Mafric	L6	■■■■■■□□□□■■■■■■■■■■■■■■■■■■■■■■■■■■■■□■■■■	West Africa II		31	1.20
Mafric	L6	■■■■■■□□■□■■■■■■■■■■■■■■■■■■■■■■■■■■■■□■■■■	West Africa II		3	0.12
Mafric	L6	■■■■■■□□□■■■■■■■■■■■■■■■■■■■■■■■■■■■■□■□□□	West Africa II		3	0.12
Mafric	L6	■■■■■■■■■□■■■□■■■■■■■■■■■■■■■■■■■■■■■■□■■■■	West Africa II	orphan	1	0.10
Mafric	L6	■■■■■■□□□■■■■□■■■■■■■□□□■■□□■■■■■■■■■■□■■■■	West Africa II		3	0.12
Mafric	L6	■■■■■■□□■■■■■■■■■■■■■■■■■■■■■■■■■■■■■■■■■	West Africa II		3	0.12

Specie	SNP	Spoligotyping profile	Sub lineage	SIT	No	%
Mafric	L6	■■■■■■□□□■■■■■■■■■■■■□□□■■■■■■■■■■■■■■□■■□□	West Africa II	orphan	1	0.04
Mafric	L6	■■■■■■□□□□■■■□■■■■■■■□□□■■■■■■■■■■■■■■□■■■■	West Africa II		8	0.30
Mafric	L6	■■■■■■■■■□■■■■■■■■■■■■■■■■■■■■■■■□■■□■■■■	West Africa II	orphan	1	0.04
Mafric	L6	■■■■■■□□□□□□■■■■■■■■■□□□■■■■■■■■■■■■■■□■■■■	West Africa II	orphan	1	0.04
Mafric	L6	■■■■■■□□□■■■■■■■■■■■■■■■■■■□■■■■■■■■■■□■■■■	West Africa II		7	0.27
Mafric	L6	■■■□□□□□□□□■■■■■■■■■■■■■■■■■■■■■■■■■□□□■■■■	West Africa II	orphan	1	0.04
Mafric	L6	■■■■■■□□□□■■■□■■■■■■■■■■■■■■■■■■■■■■■■□■■■■	West Africa II	orphan	1	0.04
Mafric	L6	■■■■■■□□□■■■■■■■■■■■■□□□■□□■■■■■■■■■■■□□■■■	West Africa II	orphan	1	0.04
Mafric	L6	■■■■■■□□□□■■■■■■■■■■■■■■■■■■■■■□□■■■■■□■■■■	West Africa II	orphan	1	0.04
Mafric	L6	■■■■■■□□■■■■■■■■■■■■■□□□■■■■■■■■■■■■■■■■■■■	West Africa II	orphan	1	0.04
Mafric	L6	■■■■■■□□□□■■■■■■■■■■■■■■■■■■■■■■■□■■□■■■■	West Africa II	orphan	1	0.04
Mafric	L6	■■■■■■□□□■■■■■■■■■■■■■■■■■■□□□■■■■■■■■□■■■■	West Africa II	orphan	1	0.04
Mafric	L6	■■■■□■□□□■■■■■■■■■■■■■□□□■■■■■■■■■■■■■□■■■■	West Africa II	orphan	1	0.04
M. bovis		■■□■■■■■□■■■■■■□■■■■■■■■■■■■■■■■■■■■■■□□□□□	BOVIS 1_BCG	482	2	0.07
M. bovis		■■□■■■■■□■■■■■■□■■■■■■■■■■■■■□■■■■■■■■□□□□□	BOVIS 1	1037	5	0.20
M. bovis		■■□■■■■■□■■■■■■□■■■■■■■■■■■■■■■■■■■■■■□□□□□	BOVIS		3	0.12
M. bovis		■■□■■□■■□■■■■■■□■■■□■■■■■■■■■□■■■■■■■■□□□□□	BOVIS		2	0.07
M. bovis		■■□■■■■□□□□□■□□□■■■■■■■■■■■■■□□□■■■■■■□□□□□	BOVIS		1	0.04
M. bovis		■■□■■□■■□■■■■■■□■■■■■■■■■■□■■■■■■■■■■■■□□□□	BOVIS		2	0.07
M. caprae		□□□□□□□□□□□□□□□□□□□■■■■■■■■■■■■■□■■■■■□■□□□	caprae		1	0.04
