# Supplementary material for: Spatio-Temporal Distribution of Mycobacterium tuberculosis Complex Strains in Ghana
Source: PLoS One. 2016 Aug 26;11(8):e0161892. doi: 10.1371/journal.pone.0161892 (PMC5001706; doi:10.1371/journal.pone.0161892)
Supplement: S8 Table — (PDF) [file pone.0161892.s008.pdf]

Table S8: Distribution of Species and Lineages of MTBC

Species	Lineages/species	Sub-Lineages	Number of Isolates (%)
MTBSS			2019 (79.1%)
	L1		36 (1.4%)
	L2		77 (3.0%)
	L3		23 (0.9%)
	L4		1883 (74.3%)*
		Cameroon	1151 (61.1%)*
		Ghana	330 (17.5%)*
		Haarlem	119 (6.3%)*
		LAM	39 (2.1%)*
		Uganda I	38 (2.0%)*
		Uganda II	5 (0.3%)*
		New-1	1 (0.1%)*
		S	3 (0.2%)*
		H37Rv-like	2 (0.2%)*
MAF			516 (20.3%)
	L5		338 (13.3%)
	L6		178 (7.0%)
Animal Strains			16 (0.7%)
	M. bovis		15 (0.6%)
	M. caprae		1 (0.0%)

*as proportion of the total number of Lineage 4 strains (1883) and not the total number of MTBC (2551)
